# U wave mimicking long QT interval: the role of lead selection, repeated electrocardiograms, and premature atrial complexes

**DOI:** 10.1093/ehjcr/ytaf264

**Published:** 2025-05-27

**Authors:** Fawzi Kerkouri, Florent Le Ven, Sarah Kerkouri, Jacques Mansourati

**Affiliations:** Department of Cardiology, University Hospital of Brest, Boulevard Tanguy Prigent, 29200 Brest, France; Université de Bretagne Occidentale (Univ Brest), Inserm, ORPHY EA 4324, 29200 Brest, France; Department of Cardiology, University Hospital of Brest, Boulevard Tanguy Prigent, 29200 Brest, France; Université de Bretagne Occidentale (Univ Brest), CHU de Brest, 29200 Brest, France; Department of Cardiology, University Hospital of Brest, Boulevard Tanguy Prigent, 29200 Brest, France; Université de Bretagne Occidentale (Univ Brest), Inserm, ORPHY EA 4324, 29200 Brest, France

## Case presentation

**Figure 1:**
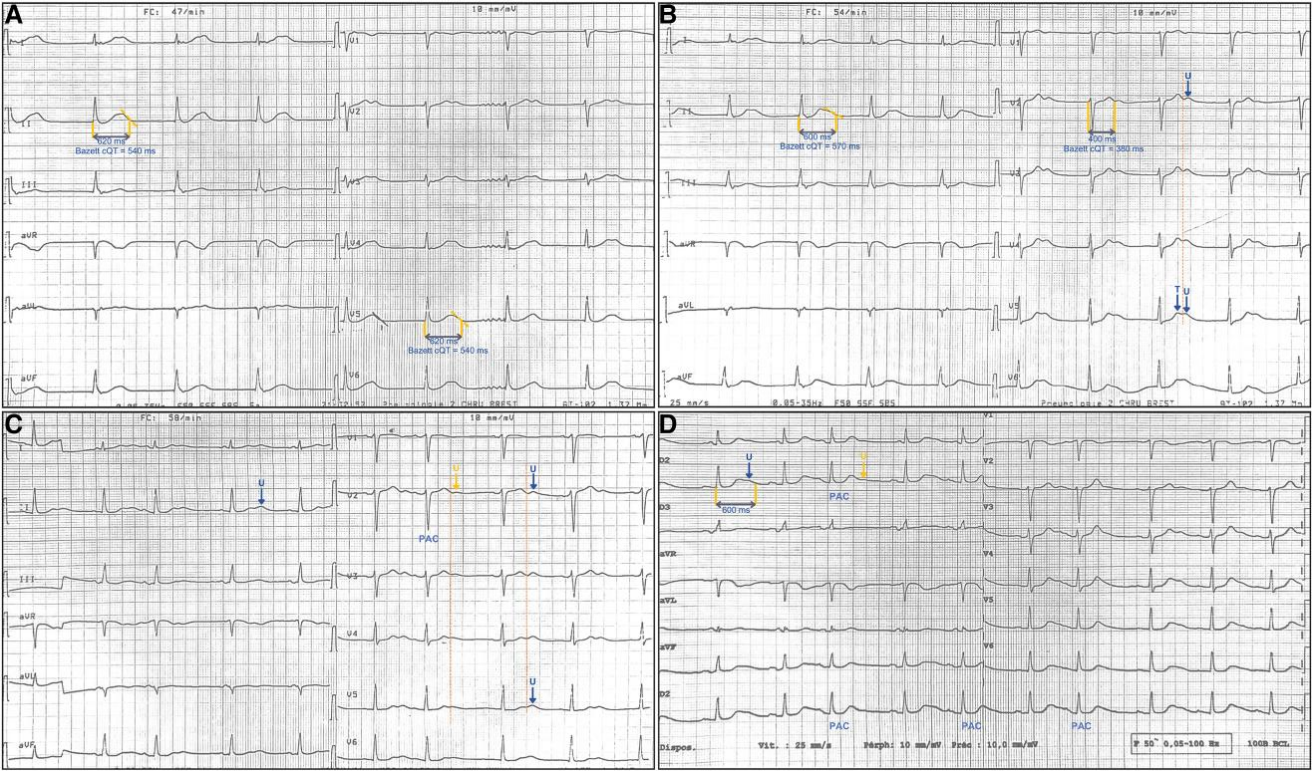
(*A*) Baseline ECG at 47/min showing prolonged QT interval in DII/V5. (*B*) At 54/min, a double wave appeared in V5, suggesting potential U wave fusion. (*C*) At 58/min, the second wave’s amplitude varied with heart rate and premature atrial complexes, while the T wave remained unchanged, supporting its identification as a U wave. (*D*) At 62/min, the second wave amplitude decreased, further reduced by premature atrial complexes, confirming the U wave’s sensitivity to cycle length. QT interval was measured in V2, where T and U waves were clearly separated.

A 78-year-old woman with hypertension, paroxysmal atrial fibrillation treated by amiodarone (200 mg/day), and idiopathic pulmonary hypertension was admitted for cardiac decompensation. Her baseline electrocardiogram (ECG) showed sinus dysfunction and an apparently prolonged corrected QT (QTc) interval (540 ms) in leads V5 and DII (*Figure 1A*), without electrolyte disturbances or ischaemia. To determine if this represented true QT prolongation or false prolongation due to U-T wave fusion,^1^ repeated ECGs at varying heart rates were performed. At 54/min, a double wave appeared in V5, suggesting a possible U wave causing false QT prolongation (*Figure 1B*). At 58/min, the second wave amplitude exceeded the first and notably decreased following premature atrial complexes (PACs). This cycle length variation supports its identification as a U wave rather than a bifid T wave (*Figure 1C*).^2^ At 62/min after mild exercise, the second wave amplitude further diminished, particularly after PACs, reinforcing its identification as a U wave (*Figure 1D*). As recommended leads (V5/II) showed persistent U-T wave fusion,^3^ measuring QT interval in these leads would erroneously suggest QT prolongation. Therefore, QT interval was accurately assessed in lead V2, where T and U waves were clearly separated, confirming a normal QT interval of 400 ms.

## Data Availability

The data supporting the findings of this case report are available from the corresponding author upon reasonable request.
